# Signals of disproportionate reporting of events supposedly attributable to COVID-19 vaccines in children and adolescents: case-non-case study, Brazil, 2022

**DOI:** 10.1590/S2237-96222026v35e20250843.en

**Published:** 2026-03-06

**Authors:** Roberta Mendes Abreu Silva, Martha Elizabeth Brasil da Nóbrega, Jadher Percio, Adriano Ferreira Martins, Silvio Luis Rodrigues Almeida, Victor Bertollo Gomes Porto

**Affiliations:** 1 Ministério da Saúde, Programa de Treinamento em Epidemiologia Aplicada aos Serviços do Sistema Único de Saúde, Brasília, DF, Brasil; 2 Ministério da Saúde, Departamento do Programa Nacional de Imunizações, Brasília, DF, Brasil; 3 Secretaria de Estado da Saúde do Distrito Federal, Brasília, DF, Brasil

**Keywords:** Vaccines, Pharmacovigilance, Adverse Event, COVID-19 Vaccines, Data Mining., Vacunas, Farmacovigilancia, Eventos adversos, Vacunas contra la covid-19, Minería de Datos.

## Abstract

**Objectives:**

To trace and describe signals of disproportionate reporting for COVID-19 vaccines in children and adolescents in Brazil, based on the analysis of reports of events supposedly attributable to vaccination or immunization (ESAVI), comparing the signals detected with events described in the package inserts.

**Methods:**

Case-non-case study based on the ESAVI reported in the e-SUS Notifica system in 2022, calculated by the reporting *odds ratio* (ROR) and information component (IC). Signal definition criteria were greater than or equal to three notifications, ROR greater than one, or credibility interval (CI) _025_ greater than zero.

**Results:**

In 2022, there were 225,693 reports, of which 26,128 were vaccine-event pairs in children and adolescents, mostly non-serious events (84.6%). We traced 39 signals of disproportionate reporting for the Sinovac vaccine and 53 signals for the Pfizer-BioNTech vaccine, including signals not described in the package insert. For Sinovac, the terms not described in the package insert were: dysarthria (ROR 23.44; IC 1.73), epistaxis (ROR 5.22; IC 1.49), ocular pruritus (ROR 4.33; IC 1.19) and muscle weakness (ROR 3.75; IC 1.19). For Pfizer-BioNTech, lymph node pain (ROR 26.74; IC 1.49), epistaxis (ROR 9.65; IC 1.76), 1st degree atrioventricular block (ROR 9.36; IC 1.44), hypersomnia (ROR 7.49; IC 1.36) and chest pain (ROR 6.03; IC 1.63).

**Conclusion:**

The analysis traced signals of disproportionate reporting of ESAVI in children and adolescents, including events not described in the package insert, which should be considered hypothesis generators. We note the need for further epidemiological studies to investigate the findings.

## Resumen

**Objetivos:**

Identificar y describir señales de notificación desproporcionada de vacunas contra la covid-19 en niños y adolescentes brasileños desde el análisis de notificaciones de eventos supuestamente atribuibles a la vacunación o inmunización (Esavi) al comparar las señales detectadas con los eventos descritos en los prospectos.

**Métodos:**

Estudio de casos/no casos basado en los Esavi notificados en el sistema e-SUS Notifica en 2022, calculado por la razón de probabilidades notificada (*reporting odds ratio*, ROR) y el componente de información (*information component*, IC). Los criterios para definir una señal fueron mayores o iguales a 3 notificaciones, ROR mayor a 1 o IC_025_ mayor a 0.

**Resultados:**

En 2022 hubo 225.693 notificaciones; de estas 26.128 fueron pares vacuna-evento en niños y adolescentes; con el 84,6% de eventos no graves. Se identificaron 39 señales de notificación desproporcionada para la vacuna Sinovac y 53 para Pfizer-BioNTech, incluidas señales no descritas en el prospecto. Para Sinovac, los términos no descritos en el prospecto fueron: disartria (ROR 23,44; IC 1,73), epistaxis (ROR 5,22; IC 1,49), prurito ocular (ROR 4,33; IC 1,19) y debilidad muscular (ROR 3,75; IC 1,19). Para Pfizer-BioNTech, dolor en los ganglios linfáticos (OR 26,74; IC 1,49), epistaxis (ROR 9,65; IC 1,76), bloqueo auriculoventricular de primer grado (ROR 9,36; IC 1,44), hipersomnia (ROR 7,49; IC 1,36) y dolor torácico (ROR 6,03; IC 1,63).

**Conclusión:**

Se identificó señales de notificaciones desproporcionadas de Esavi en esta población, incluidos eventos no descritos en el prospecto que generarán hipótesis. Es necesario realizar más estudios epidemiológicos para investigar los hallazgos.

**Ethical aspects**

**This research respected the ethical principles, having obtained the following approval data:**

Research Ethics Committee: National Committee for Research Ethics

Opinion number: 7.269.074

Approval date: 11/12/2024

Certificate of Submission to Ethical Appraisal: 82083424.1.0000.0008

Informed consent: Not applicable.

## Introduction

The 2022 epidemiological profile indicated a scenario of introduction of new variants of the SARS-CoV-2 virus and a high number of hospitalizations for severe acute respiratory syndrome due to COVID-19 in children and adolescents, with 783 confirmed deaths (1). Thus, COVID-19 vaccination in children was an important preventive resource, since complications and deaths related to long covid disease and multisystem inflammatory syndrome usually occur two to four weeks after SARS-CoV-2 infection (1).

Considering this scenario, COVID-19 vaccination strategies were expanded to the pediatric population with the vaccines Comirnaty (Pfizer-BioNTech), for children aged from six months, and CoronaVac (Sinovac/Butantan), for children aged from 3 years. The safety profile for COVID-19 vaccines in children is under continuous assessment through pharmacovigilance (2,3). However, factors such as vaccine hesitancy reduce adherence to vaccination and raise questions about events supposedly attributable to vaccination or immunization (ESAVI) that could not be initially described in clinical trials and require further characterization (2).

Monitoring reports in the e-SUS Notifica information system provides both the identification of ESAVI occurrence patterns in the population and the detection of safety signals (4-6). In this context, statistical methods and computational techniques can be applied in data sets to trace events, patterns, correlations and predict ESAVI-related outcomes (4). Several statistical measures for the detection of safety signals have been proposed in the literature, including the analysis of disproportionality (5,6).

The set of spontaneous reports allows for the review of cases, the assessment of case series and the application of simple quantitative filters, thus having high clinical informative value for the detection of safety signals in pharmacovigilance. These traditional methods are the basis of safety signal detection activities; however, the need to obtain knowledge about drug safety in a systematic and auditable manner in Brazil leads to the application of more complex methods for this purpose (5-7).

Considering the need for an in-depth assessment of ESAVI related to COVID-19 vaccines in children and adolescents, and the fact that disproportionality analysis has not yet been fully implemented for detection of signals of disproportionate reporting in Brazil, this study aims to trace and describe signals of disproportionate reporting for COVID-19 vaccines in children and adolescents in Brazil, based on the analysis of ESAVI reports, in addition to comparing the signals of disproportionate reporting detected with the events described in the package inserts of these immunobiological agents.

## Methods

### Design

Observational case-non-case study, measured by disproportionality analysis specifically focused on the analysis of pharmacovigilance databases (8). This analysis was applied, in the Brazilian context, to the reporting of ESAVI in the e-SUS Notifica information system, comparing the findings with the events described in the vaccine package inserts.

### Background

The COVID-19 pandemic resulted in the emergency introduction of vaccines for different age groups, including children and adolescents, with the aim of reducing severe cases, hospitalizations and deaths. In Brazil, COVID-19 vaccination in children aged under 18 years was incorporated into the National Immunization Program (*Programa Nacional de Imunização*, PNI) from 2021, with a progressive expansion of age indications. In parallel, pharmacovigilance systems play a key role in tracing ESAVI, especially serious, rare and unexpected ones, which are often not observed in clinical studies. Disproportionality analysis is a widely used statistical tool to assess potential signals of disproportionate reporting in spontaneous reporting databases. 

### Participants 

Vaccinated children and adolescents (aged 6 months to less than 18 years) who had at least one report of ESAVI, from January to December 2022, in Brazil.

### Definitions

ESAVI: Any undesired medical occurrence post-vaccination, not necessarily having a causal relation with the use of a vaccine or other immunobiological agent (heterologous sera and immunoglobulins). An ESAVI can be any undesirable or unintended event, i.e., symptom, disease, or abnormal laboratory finding (1,2). 

Serious ESAVI: Any clinically relevant event that (i) requires hospitalization; (ii) may compromise the patient, that is, that causes risk of death or requires immediate clinical intervention to avoid death; (iii) causes significant dysfunction and/or permanent disability; (iv) results in congenital anomaly; and (v) causes death (2). 

Non-serious ESAVI: Any event that does not meet the criteria for serious ESAVI (2). 

Report: individual record of ESAVI, which may contain one or more events and one or more vaccines administered (2).

Vaccine-ESAVI pair: Individual pairs containing only one vaccine and one event (for example, if a report contains two vaccines and three ESAVI, it will generate six [2x3] vaccine-ESAVI pairs).

Safety signal: information from one or multiple sources that suggests a new potentially causal association, or a new aspect of a known association, between an intervention and an event or set of related events, whether adverse or beneficial, considered of sufficient probability to justify a verification action (5).

### Variable

The dependent variable was the ESAVI, coded according to the medical dictionary for regulatory activities (MedDRA), categorized into five hierarchical levels of medical terms: 1) lowest level term (LLT); 2) preferred term (PT); 3) high level term (HLT); 4) high level group term (HLGT); 5) System and organ class (SOC) (9). The events are notified as LLT and, for this study, were aggregated by PT and their respective SOC.

COVID-19 CoronaVac (Sinovac) (10) and Comirnaty (Pfizer-BioNTech) (11) vaccines versus other calendar vaccines were considered as independent variables. It should be noted that the Janssen (Janssen-Cilag) and Oxford/Covishield (Fiocruz and AstraZeneca) vaccines were not evaluated, since they were not authorized for use in the pediatric population in Brazil.

### Data source and measurement

ESAVI reports were extracted through the e-SUS Notifica online system, updated on Dec 12, 2022. The e-SUS Notifica gathers individual reports of ESAVI cases submitted by health care professionals from all over the country. E-SUS Notifica is a national health information system in Brazil, which provides public and anonymized data through the OpenDataSUS platform (12).

The measurement of the results considered a 2x2 contingency table for calculations of measures of disproportionality based on vaccine-ESAVI pairs (Table 1).

**Table 1:** Contingency to assess the disproportionality of reports in e-SUS Notifica. Brazil, 2022

	Case		Non-case	Total
	ESAVI with the PT of interest		ESAVI with other PT	
COVID-19 vaccine	a		b	a+b
Other vaccines	c		d	c+d
Total reports	a+c		b+d	

a Number of reports of events supposedly attributable to vaccination or immunization (ESAVI) with the preferred term (PT) of interest for the COVID-19 vaccine investigated; ^b^Number of reports with the COVID-19 vaccine of interest and with other PT reported; ^c^Number of reports of ESAVI with the PT of interest for other COVID-19 vaccines reported; ^d^Number of reports with other COVID-19 vaccines and other PT reported.

### Bias control

As it is a spontaneous reporting system, e-SUS Notifica is subject to underreporting, especially of mild and self-limited events. Notoriety bias, characterized by increased reporting of serious or widely publicized events, was mitigated by requiring a minimum of three vaccine-ESAVI pairs per event. In addition, each event was analyzed at the level of pair with a single vaccine, minimizing possible confounders related to the co-administration of immunobiological agents. Finally, as disproportionality analysis does not allow for estimating absolute risk due to the absence of population denominators, two complementary metrics were applied to increase the robustness of the findings.

### Study size

We included all reports of ESAVI in children and adolescents (aged 6 months to <18 years) registered in the e-SUS Notifica system during the study period, which eliminated the need for sample calculation, ensuring the total analysis of the data for the period.

### Statistical analysis

For data analysis, the database was initially extracted and disaggregated into individual vaccine-ESAVI pairs, as defined in the topic “Definitions.” We excluded vaccine-ESAVI pairs that had an inconsistent temporal relation between vaccination and event onset (event initiated before vaccination or more than 42 days after it) (2), duplicate records, “immunization error” with no associated event and individuals younger than 6 months or older than 18 years.

The measures used were the reporting odds ratio (ROR) (4) and the information component (IC) (13-16), calculated using the Bayesian confidence propagation neural network (BCPNN) technique. Binomial logistic regression was used to calculate the ROR and the respective 95% confidence interval (95%CI). The criteria to define a signal of disproportionate reporting were: lower limit of the 95%CI>1 for the ROR or credible interval (CI)_025_>0 for the IC, and number of cases ≥3 per vaccine-ESAVI pair (13).

The signals of disproportionate reporting identified by both methods were compared with the ESAVI described in the package inserts of the immunobiological agents analyzed. The software used for analysis was R: RStudio version 4.2.2, using the PhViD package to calculate the BCPNN.

## Results

**Figure 1:**
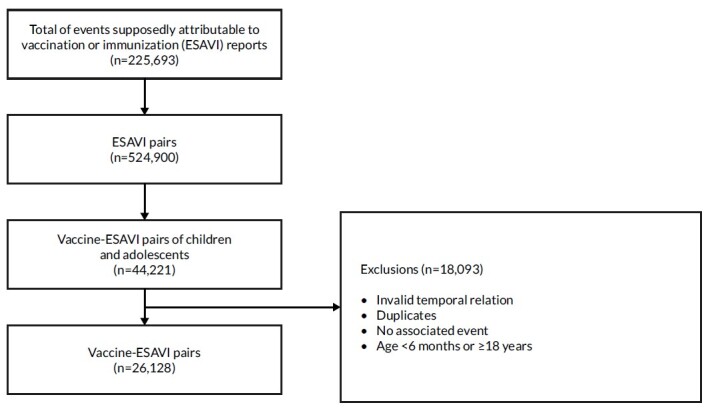
Selection of pairs of vaccine-event supposedly attributable to vaccination or immunization in children and adolescents. Brazil, 2022 (n=225,693)

A total of 225,693 reports of ESAVI were recorded. After disaggregation, 524,900 individual vaccine-ESAVI pairs were traced in the database, of which 44,221 referred to children and adolescents. After applying the exclusion criteria, 26,128 pairs remained in the database. Of these, 9.7% corresponded to the Sinovac vaccine, 15.8% to the Pfizer vaccine and 74.6% to other vaccines (non-COVID-19 vaccines) (Figure 1 ).

Most reports were classified as non-serious ESAVI (84.6%), in the age group between 6 months and 4 years of age (68.4%). The reports were mostly related to the first dose (43.8%) and presented a balanced distribution between the sexes (52.1% male). When observed by vaccine, the age group with the highest frequency of reporting, for both Sinovac and Pfizer, was between 5 and 11 years (Table 2).

**Table 2:** Absolute number and percentage of events supposedly attributable to vaccination or immunization (ESAVI) by sex, age group, severity and dose applied, according to type of vaccine (Sinovac, Pfizer and other vaccines). Brazil, 2022 (n=26,128)

Variables	Total		Sinovac		Pfizer		Other vaccines
	n (%)		n (%)		n (%)		n (%)
**Sex**							
Male	13,605 (52.1)		1,192 (47.3)		2,172 (52.7)		10,241 (52.6)
Female	12,523 (47.9)		1,330 (52.7)		1,948 (49.3)		9,245 (47.4)
**Age group (years)**							
≥6 months-4	17,869 (68.4)		327 (13.0)		113 (2.7)		17,429 (89.4)
5-11	6,570 (25.1)		1,991 (79.0)		3,142 (76.3)		1,437 (7.4)
12-17	1,689 (6.5)		201 (8.0)		865 (21.0)		623 (3.2)
**Severity classification**							
Serious ESAVI	4,028 (15.4)		223 (8.8)		421 (10.2)		3,384 (17.4)
Non-serious ESAVI	22,100 (84.6)		2,299 (91.2)		3,699 (89.8)		16,102 (82.6)
**Doses**							
1st	11,455 (43.8)		1,976 (78.4)		2,857 (69.3)		6,622 (34.0)
2nd	5,906 (22.6)		432 (17.1)		979 (23.8)		4,495 (23.1)
Others	8,767 (33.6)		114 (4.5)		284 (6.9)		8,369 (42.9)

We identified 39 signals of disproportionate reporting for the Sinovac vaccine, of which 18 were detected exclusively by the ROR and 21 coincided between the ROR and the IC. For the Pfizer vaccine, we detected 53 signals of disproportionate reporting: 37 signals at the intersection between ROR and IC, 15 exclusively by ROR and 1 by IC. Of the total signals identified, 11 (52.4%; 11 of 21) were not described in the Sinovac vaccine package insert and 18 (48.7%; 18 of 37) were not described in the Pfizer vaccine package insert.

For SOC class “General disorders and conditions at the administration site,” only chills were not described for the pediatric population in the package insert of the Sinovac vaccine (10), being present only for adults (≥18 years). For the Pfizer vaccine (11), chest pain was not described in the package inserts for any age group and was 6.03 times more reported with the Pfizer vaccine than with others (Table 3).

In the “Nervous system disorders” SOC, the term dysarthria was not present in the package insert of the Sinovac vaccine for any age group (10); for the Pfizer vaccine, only the term hypersomnia was not described in the package insert (11) (Table 3).

Ten signals of disproportionate reporting were identified for the “Respiratory, thoracic and mediastinal disorders” SOC. Of these, 2 terms (epistaxis and sneezing) were not described in the Sinovac vaccine package insert for any age group (10) and none of the 10 terms were described in the Pfizer vaccine package insert (11) (Table 3).

For “Eye Disorders,” 9 terms were identified. For the Sinovac vaccine, the terms “ocular hyperemia” and “pruritus” were not described in the package insert (10) and, for the Pfizer vaccine, only “eye irritation” and “blurry vision” were not described in the package insert (11) (Table 3).

**Table 3:** System and organ class (SOC) and preferred term (PT) of serious events supposedly attributable to vaccination (S), reporting odds ratio (ROR) and its 95% confidence interval (95%CI), information component (IC) and lower limit of the credible interval (CI_025_) according to Sinovac and Pfizer vaccines in children and adolescents. Brazil, 2022 (n=225,693) - part 1

System and organ class/preferred term	n^a^	Sinovac							Pfizer					
		n^b^	S^c^(%)	ROR	95%CI	IC	CI_025_		n^b^	S^c^(%)	ROR	95%CI	IC	CI_025_
**General disorders and conditions in the administration site**														
Asthenia	94	29	6.9	4.21	2.71; 6.54	1.54	1.04		34	14.7	3.04	2.00; 4.64	1.12	0.65
Chills/shiver	88	16	0.0	2.09	1.21; 3.59	0.79	0.15		44	0.0	5.39	3.54; 81.90	1.57	1.14
Axillary pain	7	0	-	-	-	-	-		7	0.0	-	-	1.82	0.60
Chest pain	70	20	10.0	3.77	2.24; 6.34	-	-		37	32.4	6.03	3.77; 9.66	1.63	1.15
Face oedema	42	0	-	-	-	-	-		13	30.8	2.40	1.25; 4.62	0.81	0.07
Localized edema	12	4	0.0	4.69	1.41; 15.57	-	-		0	-	-	-	-	-
Fatigue	148	31	9.7	2.50	1.68; 3.72	1.04	0.57		34	8.8	1.60	1.09; 2.35	0.50	0.05
Malaise	69	23	0.0	4.71	2.85; 7.79	-	-		25	0.0	3.05	1.86; 4.98	1.09	0.54
Abnormal sensation	27	6	0.0	2.68	1.08; 6.64	-	-		0	-	-	-	-	-
**Nervous system disorder**														
Headache	789	207	3.4	3.54	3.00; 4.17	1.43	1.24		375	2.9	5.22	4.52; 6.03	1.58	1.43
Drop attack	4	0	-	-	-	-	-		3	33.3	16.04	1.67; 154.16	-	-
Dysarthria	7	5	60.0	23.44	4.55; 120.90	1.73	0.40		0	-	-	-	-	-
Balance disorder	8	5	80.0	15.63	3.73; 65.43	-	-		0	-	-	-	-	-
Hypersomnia	12	0	-	-	-	-	-		7	0.0	7.49	2.38; 23.61	1.36	0.26
Hypoesthesia	6	0	-	-	-	-	-		3	33.3	5.34	1.08; 26.49	-	-
Bell’s palsy	4	0	-	-	-	-	-		3	0.0	16.04	1.67; 154.16	-	-
Paresthesia	33	7	14.3	2.52	1.09; 5.82	-	-		12	41.7	3.06	1.50; 6.22	1.00	0.21
Presyncope	18	5	0.0	3.61	1.28; 10.12	-	-		7	0	3.40	1.32; 8.78	-	-
Syncope	405	67	10.4	1.88	1.44; 2.45	-	-		0	-	-	-	-	-
Dizziness	129	48	8.3	5.63	3.93; 8.07	1.85	1.44		57	8.8	4.27	3.01; 6.06	1.42	1.05
**Respiratory, thoracic and mediastinal disorders**														
Nasal congestion	47	13	0.0	3.59	1.89; 6.82	-	-		29	0.0	8.66	4.81; 15.61	1.80	1.24
Pleural effusion	4	3	100.0	28.11	2.92; 270.25	-	-		0	-	-	-	-	-
Dysphonia	35	0	-	-	-	-	-		15	13.3	4.02	2.06; 7.85	1.24	0.51
Oropharyngeal pain	150	47	2.1	4.33	3.06; 6.13	-	-		69	1.4	4.61	3.34; 6.37	1.49	1.15
Epistaxis	28	10	20.0	5.22	2.41; 11.31	1.49	0.63		18	16.7	9.65	4.45; 20.93	1.76	1.05
Sneezing	51	10	0.0	2.29	1.14; 4.57	0.82	0.01		0	-	-	-	-	-
Throat irritation	14	0	-	-	-	-	-		9	0.0	9.63	3.23; 28.76	1.55	0.56
Rhinorrhea	32	0	-	-	-	-	-		10	0.0	2.43	1.15; 5.14	-	-
Coughing	319	58	5.2	2.11	1.58; 2.81	0.88	0.54		103	7.8	2.59	2.04; 3.38	1.01	0.74
Productive cough	18	0	-	-	-	-	-		6	0.0	2.67	1.00; 7.13	-	-
**Ocular disorders**														
Eye allergy	10	0	-	-	-	-	-		4	0.0	3.56	1.01; 12.63	-	-
Eye pain	19	0	-	-	-	-	-		13	0.0	11.61	4.41; 30.56	1.74	0.90
Eye edema	25	0	-	-	-	-	-		8	0.0	2.52	1.09; 5.84	-	-
Eyelid edema	42	11	9.1	3.33	1.67; 6.64	-	-		0	-	-	-	-	-
Periorbital edema	15	4	25.0	3.41	1.08; 10.71	-	-		0	-	-	-	-	-
Ocular hyperemia	47	10	20.0	2.54	1.26; 5.11	0.91	0.10		0	-	-	-	-	-
Eye irritation	11	0	-	-	-	-	-		6	0.0	6.42	1.96; 21.04	1.23	0.07
Eye pruritus	19	6	33.3	4.33	1.64; 11.40	1.19	0.11		-	-	-	-	-	-
Blurry vision	14	0	0.0	5.21	1.74; 15.55	-	-		7	0.0	5.35	1.96; 15.26	1.21	0.14

a Total reports in the database; ^b^Total reports per vaccine (CoronaVac or Pfizer); ^c^Percentage of serious ESAVI; ^d^Data not applicable for analysis.

The term abdominal discomfort was not described in the package insert of the Sinovac vaccine (10) in the SOC “Gastrointestinal disorders”; while the terms abdominal pain and odynophagia were not described in the package insert of the Pfizer vaccine (11) (Table 4).

Six terms were identified for “Musculoskeletal and connective tissue disorders.” Of these, 2 (muscle weakness and myalgia) were detected by both methods for the Sinovac vaccine and 5 (arthralgia, extremity pain, back pain, muscle weakness and myalgia) for the Pfizer vaccine (Table 4).

In total, we identified 4 terms for “Skin and subcutaneous tissue disorders” and 3 for “Infections and infestations.” Of these, 2 (pruritus and rhinitis) were detected by both methods for the Sinovac vaccine, and 3 (pruritus, urticaria and rhinitis) for the Pfizer vaccine (Table 4).

In the SOC “Psychiatric disorders,” for the Sinovac vaccine, the term anxiety was identified by both analyses and is not described in the package insert (10). For the Pfizer vaccine, in the SOC “Blood and lymphatic system disorders” and “Investigations,” 3 terms were identified (lymph node pain, lymphadenopathy and decreased blood pressure), and only lymph node pain was not described in the package insert (11) (Table 4).

**Table 4:** System and organ class (SOC) and preferred term (PT) of serious events supposedly attributable to vaccination (S), reporting odds ratio (ROR) and its 95% confidence interval (95%CI), information component (IC) and lower limit of the credible interval (CI_025_) according to Sinovac and Pfizer vaccines in children and adolescents. Brazil, 2022 (n=225,693) - part 2

System and organ class/preferred term	n^a^	Sinovac							Pfizer					
		n^b^	S^c^(%)	ROR	95%CI	IC	CI_025_		n^b^	S^c^(%)	ROR	95%CI	IC	CI_025_
**Gastrointestinal disorders**														
Abdominal discomfort	313	62	12.9	2.35	1.77; 3.11	1.00	0.67		0	-	-	-	-	-
Diarrhea	434	66	0.0	1.70	1.30; 2.21	0.63	0.32		95	2.1	1.51	1.20; 1.90	0.46	0.19
Abdominal pain	703	143	4.9	2.47	2.05; 2.99	1.06	0.84		180	12.8	1.88	1.58; 2.23	0.69	0.49
Lip edema	5	0	-	-	-	-	-		3	33.3	8.02	1.34; 48.00		
Nausea	257	63	0.0	3.09	2.32; 4.12	-	-		101	2.0	3.52	2.73; 4.53	1.29	1.01
Odynophagia	12	0	-	-	-	-	-		8	0.0	10.70	3.22; 35.56	1.54	0.48
Vomiting	981	117	0.9	1.28	1.05; 1.56	-	-		0	-	-	-	-	-
**Musculoskeletal and connective tissue disorders**														
Arthralgia	27	0	-	-	-	-	-		10	0.0	3.15	1.44; 6.88	0.98	0.12
Pain in extremities	186	0	-	-	-	-	-		46	6.5	1.76	1.26; 2.46	0.61	0.22
Backache	16	0	-	-	-	-	-		8	12.5	5.35	2.01; 14.26	1.25	0.25
Pain in the neck	6	0	-	-	-	-	-		4	25	10.69	1.96; 58.40	-	-
Muscular weakness	28	8	25.0	3.75	1.65; 8.53	1.19	0.26		10	40.0	2.97	1.37; 6.44	0.94	0.08
Myalgia	290	57	5.3	2.23	1.73; 3.11	0.99	0.64		123	3.3	4.02	3.18; 5.09	1.40	1.14
**Skin and subcutaneous tissue disorders**														
Angioedema	81	0	-	-	-	-	-		20	0.3	1.76	1.06; 2.91	-	-
Pruritus	596	112	6.3	2.22	1.80; 2.74	0.94	0.69		122	4.9	1.39	1.13; 1.70	0.37	0.13
Cold sweat	5	3	0.0	14.06	2.35; 84.16	-	-		0	-	-	-	-	-
Urticaria	240	0	-	-	-	-	-		55	9.1	1.60	1.18; 2.16	0.51	0.16
**Infections and infestations**														
Pharyngitis	6	0	-	-	-	-	-		4	0.0	10.69	1.96; 58.40	-	-
Pneumonia	13	0	-	-	-	-	-		5	100.0	3.34	1.09; 10.22	-	-
Rhinitis	156	35	0.0	2.73	1.87; 3.99	1.14	0.70		59	0.0	3.28	2.37; 4.54	1.21	0.85
**Disorders of the hematological and lymphatic systems**														
Lymph node pain	6	0	-	-	-	-	-		5	0.0	26.74	3.12; 228.87	1.49	0.12
Lymphadenopathy	73	0	-	-	-	-	-		41	4.9	6.90	4.34; 10.97	1.72	1.26
**Investigations**														
Decreased blood pressure	8	0	-	-	-	-	-		6	0.0	16.05	3.24; 79.53	1.51	0.27
**Psychiatric disorders**														
Anxiety	6	4	25.0	18.75	3.43; 102.41	1.53	0.08		0	-	-	-	-	-
**Heart disorders**														
First-degree atrioventricular block	11	0	-	-	-	-	-		7	14.3	9.36	2.74; 32.00	1.44	0.32
Palpitations	10	3	0.0	4.02	1.04; 15.54	-	-		6	16.7	8.02	2.26; 28.44	1.32	0.13
**Immune system disorders**														
Hypersensitivity	216	36	5.6	1.88	1.31; 2.70	0.74	0.31		53	7.5	1.75	1.28; 2.39	0.61	0.24
**Ear and labyrinth disorders**														
Vertigo	26	7	0.0	3.46	1.45; 8.23	1.08	0.10		8	25.0	2.38	1.03; 5.47	-	-

a Total reports in the database; ^b^Total reports per vaccine (CoronaVac or Pfizer); ^c^Percentage of serious ESAVI; ^d^Data not applicable for analysis.

For the Sinovac vaccine, the terms hypersensitivity and vertigo were identified by both analyses and are described in the package insert. For the Pfizer vaccine, 3 terms were identified (first-degree atrioventricular block, palpitations and hypersensitivity), and only atrioventricular block is not described in the package insert (11) (Table 3).

## Discussion

This is the first national study that presents results of ESAVI monitored by pharmacovigilance with the tracing of disproportionate reporting for COVID-19 vaccines applied to children and adolescents in Brazil. The majority of events detected for the vaccines were coincidental in both disproportionality calculations and related to respiratory, thoracic, and mediastinal disorders. Among the coincident terms, most were not described in the package insert and were notified as non-serious and self-limited ESAVI. In general, for the Sinovac vaccine, the main findings not described in the package insert were: dysarthria, epistaxis, ocular pruritus and muscle weakness. As for the Pfizer vaccine, the non-described events were lymph node pain, epistaxis, 1^st^ degree atrioventricular block, hypersomnia and chest pain.

This study presents a classic limitation of case-non-case studies in passive surveillance databases: the possibility of underreporting of ESAVI. In routine pharmacovigilance, the reporting rate represents, on average, 6% to 10% of real cases of ESAVI, with even greater underreporting for non-serious cases (17). This can make it difficult to measure the actual incidence of ESAVI and limit the sensitivity of signal detection by disproportionality analysis (18). This study may also have suffered notoriety bias, which occurs when a vaccine receives media attention about specific adverse events, thus increasing notifications of these events (19). In addition, external validity is limited by factors such as differences in vaccination schedules, reporting practices, vaccination coverage, and the structure of surveillance systems in other countries. Such limitations may influence the representativeness of the events detected, and the findings should be interpreted with caution and complemented with other sources of evidence.

Currently, there is no specific reference that determines what limits should be adopted to define signals of disproportionate reporting. The commonly used limits are established seeking to balance two issues: the possibility of generating many “false-positive signals” if the limit is too low, or of reducing the identification of “potential signals” if the limit is too high (5,20).

In general, most of the signals of disproportionate reporting identified were related to terms included in the SOC class “Respiratory, thoracic and mediastinal disorders”, namely: nasal congestion, oropharyngeal pain, throat irritation, sneezing, coughing and dysphonia. When considering these potential events, it is important to note that, in 2022, the highest number of doses administered was observed concomitantly with periods of higher incidence of COVID-19 in this population. These signals of disproportionate reporting are probably related to COVID-19 cases and not to vaccines (21).

The signals of disproportionate reporting identified for the Sinovac vaccine were distributed among different SOC and PT) evaluated, namely: chills, dysarthria, epistaxis, sneezing, ocular hyperemia, ocular pruritus, abdominal discomfort, muscle weakness, rhinitis and vertigo. Although epistaxis was identified as a signal of disproportionate reporting for both vaccines evaluated, hypotheses were raised relating it to complications of the nasal swab collection technique for COVID-19 rRT-PCR (22). In addition, it is worth noting that epistaxis has a higher incidence between 7 and 13 years of age, and that spontaneous bleeding, associated with digital trauma, are the most common causes of nasal bleeding in childhood, usually of a benign character (23).

Dysarthria was a term identified as a signal of disproportionate reporting and refers to a group of disorders resulting from changes in muscle control of speech mechanisms, due to damage to the central or peripheral nervous system. There is wide subjective variability in the assessment of dysarthria among health care professionals (24). Currently, there is no evidence to establish a causal relation between dysarthria and the evaluated vaccine; however, its detection indicates the need for an in-depth evaluation of the cases to better understand the event.

When observing the signals of disproportionate reporting by type of vaccine, it is possible to identify that for the Pfizer vaccine the term “chest pain,” although not specifically described in the vaccine package insert (11), may be a symptom of reactions described as myocarditis (25,26).

Another term identified for the Pfizer vaccine was “first-degree atrioventricular block,” detected by both measures of disproportionality and not described in the immunobiological agent package insert (11). It is noted, however, that large-scale population studies conducted in England and Israel found that the Pfizer vaccine was not associated with a higher risk of cardiac arrhythmias or atrioventricular blocks (26,27). On the other hand, SARS-CoV-2 infection was associated with an increased risk of several cardiac adverse events, such as myocardial infarction, arrhythmias, myocarditis and pericarditis (26,27).

The identification of two signals of disproportionate reporting with possible cardiac involvement may be related to the greater sensitivity of health care professionals to report such events, given the wide dissemination, both in scientific publications and the media, about the risk of myocarditis and pericarditis with mRNA vaccines (25).

Among the results, we observed terms whose biological plausibility with the vaccine is limited or non-existent, which reinforces the need for critical analysis as to the quality of the reports. Part of these findings could be justified by the occurrence of coding errors, since the medical dictionary for regulatory activities (MedDRA) was implemented in the ESAVI reporting system in 2021, and training on coding by the medical dictionary is still ongoing.

The two measures of disproportionality provided more accurate estimates, especially for relations with few reported cases. A previous study indicates that, as the number of reports of a vaccine-ESAVI pair increases, the methods tend to present similar results (28). Notably, the frequentist ROR method generates more false-positive signals of disproportionate reporting, but less false-negatives, while the IC minimize false-positive relations (28). Some authors indicate that ROR would perform better than other techniques with regard to early detection of signals (28,29).

In Brazil, the detection of signals of disproportionate reporting by quantitative methods is still an underutilized tool and is currently not fully implemented in the routine of the National Immunization Program (PNI). Thus, continuing to monitor and manage signals of disproportionate reporting within the PNI is of paramount importance, in order to provide scientific evidence about the events reported in a systematic and auditable manner, which will contribute to a safe vaccination and support strategic discussions for reducing vaccine hesitancy.

The results of this study reflect the profile of spontaneous reports of ESAVI for COVID-19 vaccines administered to children and adolescents in Brazil during 2022. It was possible to identify and describe the signals of disproportionate reporting. It is noted that quantitative analyses of passive surveillance data, such as those presented here, have as their main objective the raising of hypotheses by the scientific community, not being adequate for causal inference (5), which indicates the need for further epidemiological studies to investigate each signal of disproportionate reporting pointed out. Although the results do not allow for direct generalizations, they can provide comparative inputs to others for populations with similar characteristics in contexts of national immunization programs that use passive pharmacovigilance systems. Finally, there is a need for improvement in the ESAVI coding process with the use of the medical dictionary for regulatory activities by reporting professionals in Brazil.

## Data Availability

Aggregated and anonymized data can be accessed athttps://doi.org/10.48331/SCIELODATA.5XCF1B
