# Supplementary material for: Signals of disproportionate reporting of events supposedly attributable to COVID-19 vaccines in children and adolescents: case-non-case study, Brazil, 2022
Source: Epidemiol Serv Saude. 2026 Mar 6;35:e20250843. doi: 10.1590/S2237-96222026v35e20250843.en (PMC12969790; doi:10.1590/S2237-96222026v35e20250843.en)
Supplement: Translation

# Translation: Sinais de notificação desproporcional de eventos supostamente atribuíveis às vacinas covid-19 em crianças e adolescentes: estudo caso-não-caso, Brasil, 2022

**DOI:** 10.1590/S2237-96222026v35e20250843.pt

**Authors:** Roberta Mendes Abreu Silva, Martha Elizabeth Brasil da Nóbrega, Jadher Percio, Adriano Ferreira Martins, Silvio Luis Rodrigues Almeida, Victor Bertollo Gomes Porto

**Affiliations:** 1 Ministério da Saúde, Programa de Treinamento em Epidemiologia Aplicada aos Serviços do Sistema Único de Saúde, Brasília, DF, Brazil; 2 Ministério da Saúde, Departamento do Programa Nacional de Imunizações, Brasília, DF, Brazil; 3 Secretaria de Estado da Saúde do Distrito Federal, Brasília, DF, Brazil

**Keywords:** Vacinas, Farmacovigilância, Eventos Adversos, Vacinas contra Covid-19, Mineração de Dados.

## Abstract

**Objetivos:**

Identificar e descrever sinais de notificação desproporcional das vacinas contra a covid-19 em crianças e adolescentes no Brasil, a partir da análise das notificações de eventos supostamente atribuíveis à vacinação ou imunização (Esavi), comparando os sinais detectados com eventos descritos nas bulas.

**Métodos:**

Estudo caso-não-caso baseado nos Esavi notificados no e-SUS Notifica em 2022, calculado pela razão de chances reportada (*reporting odds ratio*, ROR) e componente de informação (*information component*, IC). Os critérios para definição de sinal foram maiores ou iguais a 3 notificações, ROR maior que 1 ou intervalo de credibilidade (IC)_025_ maior que 0.

**Resultados:**

Em 2022, foram registradas 225.693 notificações, destas 26.128 foram pares vacina-evento em crianças e adolescentes, sendo 84,6% dos eventos não grave. Foram identificados 39 sinais de notificação desproporcional para a vacina Sinovac e 53 sinais para a vacina Pfizer-BioNTech, incluindo sinais não descritos em bula. Para Sinovac, os termos não descritos em bula foram: disartria (ROR 23,44; IC 1,73), epistaxe (ROR 5,22; IC 1,49), prurido ocular (ROR 4,33; IC 1,19) e fraqueza muscular (ROR 3,75; IC 1,19). Para Pfizer-BioNTech, dor em linfonodo (ROR 26,74; IC 1,49), epistaxe (ROR 9,65; IC 1,76), bloqueio atrioventricular de 1º grau (ROR 9,36; IC 1,44), hipersonia (ROR 7,49; IC 1,36) e dor torácica (ROR 6,03; IC 1,63).

**Conclusão:**

A análise identificou sinais de notificações desproporcionais de Esavi em crianças e adolescentes, incluindo eventos não descritos em bula, que devem ser considerados geradores de hipótese. Ressalta-se necessidade de estudos epidemiológicos adicionais para investigação dos achados.

**Aspectos éticos**

**Esta pesquisa respeitou os princípios éticos, obtendo os seguintes dados de aprovação:**

Comitê de ética em pesquisa: Comissão Nacional de Ética em Pesquisa 

Número do parecer: 7.269.074

Data de aprovação: 11/12/2024

Certificado de apresentação de apreciação ética: 82083424.1.0000.0008

Registro do consentimento livre e esclarecido: Não aplicável.

## Introdução

O perfil epidemiológico em 2022 apontava um cenário de introdução de novas variantes do vírus SARS-CoV-2 e elevado número de hospitalizações por síndrome respiratória aguda grave decorrente da covid-19, em crianças e adolescentes, com 783 mortes confirmadas (1). Dessa forma, a vacinação contra covid-19 em crianças foi um recurso preventivo importante tendo em vista que as complicações e os óbitos relacionados à doença covid longa e à síndrome inflamatória multissistêmica ocorrem, geralmente, duas a quatro semanas após a infecção pelo SARS-CoV-2(1).

Diante desse cenário, as estratégias de vacinação contra covid-19 foram ampliadas para o público pediátrico com as vacinas Comirnaty (Pfizer-BioNTech), para crianças a partir de seis meses de idade, e CoronaVac (Sinovac/Butantan), para crianças a partir de 3 anos. O perfil de segurança das vacinas de covid-19 em crianças está sob avaliação contínua por meio da farmacovigilância (2,3). No entanto, fatores como a hesitação vacinal enfraquecem a adesão à vacinação e levantam questionamentos sobre os eventos supostamente atribuíveis à vacinação ou imunização (Esavi) que não puderam ser inicialmente descritos nos ensaios clínicos e que precisam ser melhor caracterizados (2).

O monitoramento das notificações geradas no sistema de informação e-SUS Notifica permite tanto a identificação de padrões de ocorrência de Esavi na população quanto a detecção de sinais de segurança (4-6). Nesse contexto, é possível aplicar métodos estatísticos e técnicas computacionais em conjuntos de dados para identificar eventos, padrões, correlações e prever desfechos relacionados aos Esavi (4). Diversas medidas estatísticas para detecção de sinais de segurança foram propostas na literatura, entre elas a análise de desproporcionalidade (5,6).

O conjunto de notificações espontâneas permite a revisão de casos, a avaliação de séries de casos e a aplicação de filtros quantitativos simples, tendo, portanto, alto valor informativo clínico para a detecção de sinais de segurança em farmacovigilância. Esses métodos tradicionais são a base das atividades de detecção de sinais de segurança; no entanto, a necessidade de se obter conhecimento sobre a segurança de medicamentos de forma sistemática e auditável no Brasil impulsiona a aplicação de métodos mais complexos para essa finalidade (5-7).

Considerando a necessidade de uma avaliação aprofundada dos Esavi relacionados às vacinas de covid-19 em crianças e adolescentes, e ao fato de que a análise de desproporcionalidade ainda não foi plenamente implementada para a detecção de sinais de notificação desproporcional no Brasil, este estudo tem como objetivo identificar e descrever sinais de notificação desproporcional das vacinas contra a covid-19 em crianças e adolescentes no Brasil, a partir da análise das notificações de Esavi, além de comparar os sinais de notificação desproporcional detectados com os eventos descritos nas bulas desses imunobiológicos.

## Métodos

### Delineamento

Trata-se de estudo observacional do tipo caso-não-caso, mensurado pela análise de desproporcionalidade voltado especificamente à análise de bases de dados de farmacovigilância (8). Essa análise foi aplicada, no contexto brasileiro, nas notificações de Esavi no sistema de informação e-SUS Notifica, comparando os achados encontrados com os eventos descritos nas bulas de vacinas.

### Contexto

A pandemia de covid-19 resultou na introdução emergencial de vacinas para diferentes faixas etárias, incluindo crianças e adolescentes, com o objetivo de reduzir casos graves, hospitalizações e óbitos. No Brasil, a vacinação contra covid-19 em menores de 18 anos foi incorporada ao Programa Nacional de Imunizações (PNI) a partir de 2021, com ampliação progressiva das indicações etárias. Em paralelo, os sistemas de farmacovigilância desempenham papel fundamental para identificar Esavi, especialmente os graves, raros e inesperados, que muitas vezes não são observados em estudos clínicos. A análise de desproporcionalidade é uma ferramenta estatística amplamente utilizada para avaliar potenciais sinais de notificação desproporcional em bancos de dados de notificações espontâneas. 

### Participantes

Crianças e adolescentes vacinados (com idades entre 6 meses e menos de 18 anos) que tiveram pelo menos uma notificação de Esavi, no período de janeiro a dezembro de 2022, no Brasil.

### Definições

Esavi: qualquer ocorrência médica indesejada após a vacinação, não possuindo necessariamente uma relação causal com o uso de uma vacina ou outro imunobiológico (imunoglobulinas e soros heterólogos). Um Esavi pode ser qualquer evento indesejável ou não intencional, isto é, sintoma, doença ou achado laboratorial anormal (2). 

Esavi grave: qualquer evento clinicamente relevante que (i) requeira hospitalização; (ii) possa comprometer o paciente, ou seja, que ocasione risco de morte ou que exija intervenção clínica imediata para evitar o óbito; (iii) cause disfunção significativa e/ou incapacidade permanente; (iv) resulte em anomalia congênita; e (v) ocasione óbito (2). 

Esavi não grave: qualquer evento que não preencha os critérios dos Esavi grave (2). 

Notificação: registro individual de Esavi, podendo conter um ou mais eventos e uma ou mais vacinas administradas (2).

Par vacina-Esavi: pares individuais contendo apenas uma vacina e um evento (por exemplo, se uma notificação contém duas vacinas e três Esavi, ela gerará seis [2x3] pares vacina-Esavi).

Sinal de segurança: informação proveniente de uma ou múltiplas fontes, que sugere uma nova associação potencialmente causal, ou um novo aspecto de uma associação conhecida, entre uma intervenção e um evento ou conjunto de eventos relacionados, sejam eles adversos ou benéficos, considerada de probabilidade suficiente para justificar uma ação de verificação (5).

### Variáveis

A variável dependente foram os Esavi, codificados de acordo com dicionário médico para atividades regulatórias (*medical dictionary for regulatory activities,* MedDRA), categorizado em cinco níveis hierárquicos de termos médicos: 1) termo de nível mais baixo *(lowest level term*, LLT); 2) termo preferido (*preferred term,* PT); 3) termo de nível alto *(high level term*, HLT); 4) termo de grupo de nível alto *(high level group,* HLGT); 5) classe de sistema e órgão (*system organ class*, SOC) (9). Os eventos são notificados como LLT e, para este estudo, foram agregados por PT e suas respectivas SOC.

Foram consideradas como variáveis independentes as vacinas de covid-19 CoronaVac (Sinovac) (10) e Comirnaty (Pfizer-BioNTech) (11) versus outras vacinas do calendário. Ressalta-se que não foram avaliadas as vacinas Janssen (Janssen-Cilag) e Oxford/Covishield (Fiocruz e AstraZeneca), uma vez que estas não foram autorizadas para uso na população pediátrica no Brasil.

### Fonte de dados e mensuração

As notificações de Esavi foram extraídas por meio do sistema on-line e-SUS Notifica, com atualização em 31 de dezembro de 2022. O e-SUS Notifica reúne notificações individuais de casos de Esavi enviadas por profissionais de saúde de todo o país. O e-SUS Notifica é um sistema nacional de informação em saúde do Brasil, que disponibiliza dados públicos e anonimizados por meio da plataforma OpenDataSUS (12).

A mensuração dos resultados considerou uma tabela de contingência 2x2 para os cálculos das medidas de desproporcionalidade a partir dos pares vacina-Esavi (Tabela 1).

**Tabela 1:** Contingência para avaliação da desproporcionalidade de notificações no e-SUS Notifica. Brasil, 2022

	Caso		Não caso	Total
	Esavi com o PT de interesse		Esavi com demais PT	
Vacina covid-19	a		b	a+b
Outras vacinas	c		d	c+d
Total de notificações	a+c		b+d	

a Número de notificações de eventos supostamente atribuíveis à vacinação ou imunização (Esavi) com o termo preferido (*preferred term,* PT) de interesse para a vacina covid-19 investigada; ^b^Número de notificações com a vacina covid-19 de interesse e com demais PT reportados; ^c^Número de notificações de Esavi com o PT de interesse para outras vacinas covid-19 reportadas; ^d^Número de notificações com outras vacinas covid-19 e demais PT notificados.

### Controle de vieses

Por se tratar de um sistema de notificação espontânea, o e-SUS Notifica está sujeito à subnotificação, especialmente de eventos leves e autolimitados. O viés de notoriedade, caracterizado pela maior notificação de eventos graves ou amplamente divulgados, foi mitigado pela exigência de um número mínimo de três pares vacina-Esavi por evento. Além disso, cada evento foi analisado ao nível de par com uma única vacina, minimizando possíveis confundidores relacionados à coadministração de imunobiológicos. Por fim, como a análise de desproporcionalidade não permite estimar risco absoluto devido à ausência de denominadores populacionais, foram aplicadas duas métricas complementares para aumentar a robustez dos achados.

### Tamanho do estudo

Foram incluídas todas as notificações de Esavi em crianças e adolescentes (6 meses a <18 anos) registradas no sistema e-SUS Notifica no período do estudo, o que eliminou a necessidade de cálculo amostral, garantindo a análise total dos dados do período.

### Análise estatística

Para análise dos dados, a base foi inicialmente extraída e desagregada em pares individuais vacina-Esavi, conforme definido no tópico “Definições”. Foram excluídos os pares vacina-Esavi que apresentavam relação temporal inconsistente entre a vacinação e o início do evento (evento iniciado antes da vacinação ou mais de 42 dias após) (2), registros duplicados, “erro de imunização” sem evento associado e indivíduos com menos de 6 meses ou mais de 18 anos.

As medidas utilizadas foram a razão de chances reportada (*reporting odds ratio*, ROR) (4) e o componente de informação (*information component*, IC) (13-16), calculado por meio da técnica rede neural de propagação de confiança bayesiana (*Bayesian confidence propagation neural network*, BCPNN). Para o cálculo da ROR e respectivo intervalo de confiança de 95% (IC95%), foi utilizada regressão logística binomial. Os critérios para definir um sinal de notificação desproporcional foram: limite inferior do IC95%>1 para a ROR ou intervalo de credibilidade, IC_025_>0, para o IC, e número de casos ≥3 por par vacina-Esavi (13).

Os sinais de notificação desproporcional identificados por ambos os métodos foram comparados com os Esavi descritos nas bulas dos imunobiológicos analisados. O software utilizado para análise foi o R: RStudio versão 4.2.2, utilizando o pacote PhViD para o cálculo da BCPNN.

## Resultados

Um total de 225.693 notificações de Esavi foram registradas. Após a desagregação, foram identificados 524.900 pares individuais vacina-Esavi na base de dados, dos quais 44.221 se referiam a crianças e adolescentes. Após aplicação dos critérios de exclusão, permaneceram 26.128 pares na base de dados. Desses, 9,7% correspondiam à vacina Sinovac, 15,8% à vacina Pfizer e 74,6% a outras vacinas (vacinas não covid-19) (Figura 1).

**Figura 1:** Seleção dos pares vacina-eventos supostamente atribuíveis à vacinação em crianças e adolescentes. Brasil, 2022 (n=225.693)

A maioria das notificações foi classificada como Esavi não grave (84,6%), na faixa etária entre 6 meses e 4 anos de idade (68,4%). Os relatos estavam majoritariamente relacionados à primeira dose (43,8%) e apresentaram distribuição equilibrada entre os sexos (52,1% masculino). Quando observados por vacina, a faixa etária com maior frequência de notificações, tanto para Sinovac quanto para Pfizer, foi entre 5 e 11 anos (Tabela 2).

**Tabela 2:** Número absoluto e percentual de eventos supostamente atribuíveis à vacinação ou imunização por sexo, faixa etária, gravidade e dose aplicada, segundo tipo de vacina (Sinovac, Pfizer e outras vacinas). Brasil, 2022 (n=26.128)

Variáveis	Total		Sinovac		Pfizer		Outras vacinas
	n (%)		n (%)		n (%)		n (%)
Sexo							
Masculino	13.605 (52,1)		1.192 (47,3)		2.172 (52,7)		10.241 (52,6)
Feminino	12.523 (47,9)		1.330 (52,7)		1.948 (49,3)		9.245 (47,4)
**Faixa etária (anos)**							
≥6 meses-4	17.869 (68,4)		327 (13,0)		113 (2,7)		17.429 (89,4)
5-11	6.570 (25,1)		1.991 (79,0)		3.142 (76,3)		1.437 (7,4)
12-17	1.689 (6,5)		201 (8,0)		865 (21,0)		623 (3,2)
**Classificação de gravidade**							
Esavi grave	4.028 (15,4)		223 (8,8)		421 (10,2)		3.384 (17,4)
Esavi não grave	22.100 (84,6)		2.299 (91,2)		3.699 (89,8)		16.102 (82,6)
**Doses**							
1ª	11.455 (43,8)		1.976 (78,4)		2.857 (69,3)		6.622 (34,0)
2ª	5.906 (22,6)		432 (17,1)		979 (23,8)		4.495 (23,1)
Outras	8.767 (33,6)		114 (4,5)		284 (6,9)		8.369 (42,9)

Foram identificados 39 sinais de notificação desproporcional para a vacina Sinovac, dos quais 18 foram detectados exclusivamente pela ROR e 21 coincidiram entre o ROR e o IC. Para a vacina Pfizer, foram detectados 53 sinais de notificação desproporcional: 37 sinais na interseção entre ROR e o IC, 15 exclusivamente pela ROR e 1 pelo IC. Do total de sinais identificados, 11 (52,4%; 11 de 21) não estavam descritos na bula da vacina Sinovac e 18 (48,7%; 18 de 37) não estavam descritos na bula da vacina Pfizer.

Para a classe de SOC “Distúrbios gerais e condições no local de administração”, apenas calafrios não estava descrito para população pediátrica na bula da vacina Sinovac (10), estando presente apenas para adultos (≥18 anos). Para a vacina Pfizer (11), dor torácica não estava descrita nas bulas para nenhuma faixa etária e foi 6,03 vezes mais relatada com a vacina Pfizer do que com outras (Tabela 3).

No SOC “Distúrbios do sistema nervoso”, o termo disartria não estava presente na bula da vacina Sinovac para nenhuma faixa etária (10); para a vacina Pfizer, apenas o termo hipersonia não estava descrita na bula (11) (Tabela 3).

Dez sinais de notificação desproporcional foram identificados para o SOC “Distúrbios respiratórios, torácicos e do mediastino”. Desses, 2 termos (epistaxe e espirros) não estavam descritos na bula da vacina Sinovac para nenhuma faixa etária (10) e nenhum dos 10 termos estavam descritos na bula da vacina Pfizer (11) (Tabela 3).

Para “Distúrbios oculares”, 9 termos foram identificados. Para vacina Sinovac, os termos “hiperemia ocular” e “prurido” não estavam descritos na bula (10) e, para a vacina Pfizer, apenas “irritação ocular” e “visão turva” não estavam descritos na bula (11) (Tabela 3).

**Tabela 3:** Classe de sistema e órgão (*system organ class*, SOC) e termo preferido (*preferred term,* PT) dos eventos supostamente atribuíveis à vacinação grave (G), razão de chances reportada (*reporting odds ratio*, ROR) e seu intervalo de confiança de 95% (IC95%), componente de informação (*information component*, IC) e limite inferior do intervalo de credibilidade (IC_025_), segundo vacinas Sinovac e Pfizer em crianças e adolescentes. Brasil, 2022 (n=225.693) - parte 1

Classe de sistemas e órgãos/termo preferido	n^a^	Sinovac							Pfizer					
		n^b^	G^c^(%)	ROR	IC95%	IC	IC_025_		n^b^	G^c^(%)	ROR	IC95%	IC	IC_025_
Distúrbios gerais e condições no local de administração														
Astenia	94	29	6,9	4,21	2,71; 6,54	1,54	1,04		34	14,7	3,04	2,00; 4,64	1,12	0,65
Calafrios	88	16	0,0	2,09	1,21; 3,59	0,79	0,15		44	0,0	5,39	3,54; 81,90	1,57	1,14
Dor axilar	7	0	-	-	-	-	-		7	0,0	-	-	1,82	0,60
Dor torácica	70	20	10,0	3,77	2,24; 6,34	-	-		37	32,4	6,03	3,77; 9,66	1,63	1,15
Edema facial	42	0	-	-	-	-	-		13	30,8	2,40	1,25; 4,62	0,81	0,07
Edema localizado	12	4	0,0	4,69	1,41; 15,57	-	-		0	-	-	-	-	-
Fadiga	148	31	9,7	2,50	1,68; 3,72	1,04	0,57		34	8,8	1,60	1,09; 2,35	0,50	0,05
Mal-estar	69	23	0,0	4,71	2,85; 7,79	-	-		25	0,0	3,05	1,86; 4,98	1,09	0,54
Sensação anormal	27	6	0,0	2,68	1,08; 6,64	-	-		0	-	-	-	-	-
**Distúrbio do sistema nervoso**														
Cefaleia	789	207	3,4	3,54	3,00; 4,17	1,43	1,24		375	2,9	5,22	4,52; 6,03	1,58	1,43
Ataque de queda	4	0	-	-	-	-	-		3	33,3	16,04	1,67; 154,16	-	-
Disartria	7	5	60,0	23,44	4,55; 120,90	1,73	0,40		0	-	-	-	-	-
Distúrbio do equilíbrio	8	5	80,0	15,63	3,73; 65,43	-	-		0	-	-	-	-	-
Hipersonia	12	0	-	-	-	-	-		7	0,0	7,49	2,38; 23,61	1,36	0,26
Hipoestesia	6	0	-	-	-	-	-		3	33,3	5,34	1,08; 26,49	-	-
Paralisia de Bell	4	0	-	-	-	-	-		3	0,0	16,04	1,67; 154,16	-	-
Parestesia	33	7	14,3	2,52	1,09; 5,82	-	-		12	41,7	3,06	1,50; 6,22	1,00	0,21
Pré-síncope	18	5	0,0	3,61	1,28; 10,12	-	-		7	0	3,40	1,32; 8,78	-	-
Síncope	405	67	10,4	1,88	1,44; 2,45	-	-		0	-	-	-	-	-
Tontura	129	48	8,3	5,63	3,93; 8,07	1,85	1,44		57	8,8	4,27	3,01; 6,06	1,42	1,05
**Distúrbios respiratórios, torácicos e do mediastino**														
Congestão nasal	47	13	0,0	3,59	1,89; 6,82	-	-		29	0,0	8,66	4,81; 15,61	1,80	1,24
Derrame pleural	4	3	100,0	28,11	2,92; 270,25	-	-		0	-	-	-	-	-
Disfonia	35	0	-	-	-	-	-		15	13,3	4,02	2,06; 7,85	1,24	0,51
Dor orofaríngea	150	47	2,1	4,33	3,06; 6,13	-	-		69	1,4	4,61	3,34; 6,37	1,49	1,15
Epistaxe	28	10	20,0	5,22	2,41; 11,31	1,49	0,63		18	16,7	9,65	4,45; 20,93	1,76	1,05
Espirros	51	10	0,0	2,29	1,14; 4,57	0,82	0,01		0	-	-	-	-	-
Irritação na garganta	14	0	-	-	-	-	-		9	0,0	9,63	3,23; 28,76	1,55	0,56
Rinorreia	32	0	-	-	-	-	-		10	0,0	2,43	1,15; 5,14	-	-
Tosse	319	58	5,2	2,11	1,58; 2,81	0,88	0,54		103	7,8	2,59	2,04; 3,38	1,01	0,74
Tosse produtiva	18	0	-	-	-	-	-		6	0,0	2,67	1,00; 7,13	-	-
**Distúrbios oculares**														
Alergia ocular	10	0	-	-	-	-	-		4	0,0	3,56	1,01; 12,63	-	-
Dor ocular	19	0	-	-	-	-	-		13	0,0	11,61	4,41; 30,56	1,74	0,90
Edema ocular	25	0	-	-	-	-	-		8	0,0	2,52	1,09; 5,84	-	-
Edema de pálpebra	42	11	9,1	3,33	1,67; 6,64	-	-		0	-	-	-	-	-
Edema periorbital	15	4	25,0	3,41	1,08; 10,71	-	-		0	-	-	-	-	-
Hiperemia ocular	47	10	20,0	2,54	1,26; 5,11	0,91	0,10		0	-	-	-	-	-
Irritação ocular	11	0	-	-	-	-	-		6	0,0	6,42	1,96; 21,04	1,23	0,07
Prurido ocular	19	6	33,3	4,33	1,64; 11,40	1,19	0,11		-	-	-	-	-	-
Visão turva	14	0	0,0	5,21	1,74; 15,55	-	-		7	0,0	5,35	1,96; 15,26	1,21	0,14

a Total de notificações na base de dados; ^b^Total de notificações por vacina (CoronaVac ou Pfizer); ^c^Percentual de eventos supostamente atribuíveis à vacinação ou imunização graves; ^d^Dado não aplicável para análise.

O termo desconforto abdominal não estava descrito na bula da vacina Sinovac (10) no SOC “Distúrbios gastrintestinais”; ao passo que os termos dor abdominal e odinofagia não estavam descritos na bula da vacina Pfizer (11) (Tabela 4).

Seis termos foram identificados para “Distúrbios musculoesqueléticos e do tecido conjuntivo”. Desses, 2 (fraqueza muscular e mialgia) foram detectados por ambos os métodos para a vacina Sinovac e 5 (artralgia, dor em extremidade, dor nas costas, fraqueza muscular e mialgia) para a vacina Pfizer (Tabela 4).

No total, foram identificados 4 termos para “Distúrbios da pele e tecido subcutâneo” e 3 para “Infecções e infestações”. Desses, 2 (prurido e rinite) foram detectados por ambos os métodos para a vacina Sinovac, e 3 (prurido, urticária e rinite) para a vacina Pfizer (Tabela 4).

No SOC “Distúrbios psiquiátricos”, para a vacina Sinovac, o termo ansiedade foi identificado por ambas as análises e não está descrito na bula (10). Para a vacina Pfizer, nos SOC “Distúrbios sanguíneos e do sistema linfático” e “Investigações”, 3 termos foram identificados (dor em linfonodos, linfadenopatia e pressão arterial diminuída), e apenas dor em linfonodos não estava descrita na bula (11) (Tabela 4).

**Tabela 4:** Classe de sistema e órgão (*system organ class*, SOC) e termo preferido (*preferred term,* PT) dos eventos supostamente atribuíveis à vacinação grave (G), razão de chances reportada (*reporting odds ratio*, ROR) e seu intervalo de confiança de 95% (IC95%), componente de informação (*information component*, IC) e limite inferior do intervalo de credibilidade (IC_025_), segundo vacinas Sinovac e Pfizer em crianças e adolescentes. Brasil, 2022 (n=225.693) - parte 2

Classe de sistemas e órgãos/termo preferido	n^a^	Sinovac					Pfizer			
		n^b^	G^c^(%)	ROR	IC95%	IC	IC_025_		n^b^	G^c^(%)	ROR	IC95%	IC	IC_025_
Distúrbios gastrointestinais														
Desconforto abdominal	313	62	12,9	2,35	1,77; 3,11	1,00	0,67		0	-	-	-	-	-
Diarreia	434	66	0,0	1,70	1,30; 2,21	0,63	0,32		95	2,1	1,51	1,20; 1,90	0,46	0,19
Dor abdominal	703	143	4,9	2,47	2,05; 2,99	1,06	0,84		180	12,8	1,88	1,58; 2,23	0,69	0,49
Edema de lábio	5	0	-	-	-	-	-		3	33,3	8,02	1,34; 48,00		
Náusea	257	63	0,0	3,09	2,32; 4,12	-	-		101	2,0	3,52	2,73; 4,53	1,29	1,01
Odinofagia	12	0	-	-	-	-	-		8	0,0	10,70	3,22; 35,56	1,54	0,48
Vômito	981	117	0,9	1,28	1,05; 1,56	-	-		0	-	-	-	-	-
Distúrbios musculoesqueléticos e do tecido conjuntivo														
Artralgia	27	0	-	-	-	-	-		10	0,0	3,15	1,44; 6,88	0,98	0,12
Dor em extremidade	186	0	-	-	-	-	-		46	6,5	1,76	1,26; 2,46	0,61	0,22
Dor nas costas	16	0	-	-	-	-	-		8	12,5	5,35	2,01; 14,26	1,25	0,25
Dor no pescoço	6	0	-	-	-	-	-		4	25	10,69	1,96; 58,40	-	-
Fraqueza muscular	28	8	25,0	3,75	1,65; 8,53	1,19	0,26		10	40,0	2,97	1,37; 6,44	0,94	0,08
Mialgia	290	57	5,3	2,23	1,73; 3,11	0,99	0,64		123	3,3	4,02	3,18; 5,09	1,40	1,14
Distúrbios dos tecidos cutâneos e subcutâneos														
Angioedema	81	0	-	-	-	-	-		20	0,3	1,76	1,06; 2,91	-	-
Prurido	596	112	6,3	2,22	1,80; 2,74	0,94	0,69		122	4,9	1,39	1,13; 1,70	0,37	0,13
Suor frio	5	3	0,0	14,06	2,35; 84,16	-	-		0	-	-	-	-	-
Urticária	240	0		-	-	-	-		55	9,1	1,60	1,18; 2,16	0,51	0,16
Infecções e infestações														
Faringite	6	0	-	-	-	-	-		4	0,0	10,69	1,96; 58,40	-	-
Pneumonia	13	0	-	-	-	-	-		5	100,0	3,34	1,09; 10,22	-	-
Rinite	156	35	0,0	2,73	1,87; 3,99	1,14	0,70		59	0,0	3,28	2,37; 4,54	1,21	0,85
Distúrbios dos sistemas hematológico e linfático														
Dor no linfonodo	6	0	-	-	-	-	-		5	0,0	26,74	3,12; 228,87	1,49	0,12
Linfadenopatia	73	0	-	-	-	-	-		41	4,9	6,90	4,34; 10,97	1,72	1,26
Investigações														
Pressão arterial diminuída	8	0	-	-	-	-	-		6	0,0	16,05	3,24; 79,53	1,51	0,27
Distúrbios psiquiátricos														
Ansiedade	6	4	25,0	18,75	3,43; 102,41	1,53	0,08		0	-	-	-	-	-
Distúrbios cardíacos														
Bloqueio atrioventricular de 1º grau	11	0	-	-	-	-	-		7	14,3	9,36	2,74; 32,00	1,44	0,32
Palpitações	10	3	0,0	4,02	1,04; 15,54	-	-		6	16,7	8,02	2,26; 28,44	1,32	0,13
Distúrbios do sistema imunitário														
Hipersensibilidade	216	36	5,6	1,88	1,31; 2,70	0,74	0,31		53	7,5	1,75	1,28; 2,39	0,61	0,24
Distúrbios do ouvido e do labirinto														
Vertigem	26	7	0,0	3,46	1,45; 8,23	1,08	0,10		8	25,0	2,38	1,03; 5,47	-	-

Classe de sistemas e órgãos/termo preferido	n^a^	Sinovac							Pfizer					
		n^b^	G^c^(%)	ROR	IC95%	IC	IC_025_		n^b^	G^c^(%)	ROR	IC95%	IC	IC_025_
Distúrbios gastrointestinais														
Desconforto abdominal	313	62	12,9	2,35	1,77; 3,11	1,00	0,67		0	-	-	-	-	-
Diarreia	434	66	0,0	1,70	1,30; 2,21	0,63	0,32		95	2,1	1,51	1,20; 1,90	0,46	0,19
Dor abdominal	703	143	4,9	2,47	2,05; 2,99	1,06	0,84		180	12,8	1,88	1,58; 2,23	0,69	0,49
Edema de lábio	5	0	-	-	-	-	-		3	33,3	8,02	1,34; 48,00		
Náusea	257	63	0,0	3,09	2,32; 4,12	-	-		101	2,0	3,52	2,73; 4,53	1,29	1,01
Odinofagia	12	0	-	-	-	-	-		8	0,0	10,70	3,22; 35,56	1,54	0,48
Vômito	981	117	0,9	1,28	1,05; 1,56	-	-		0	-	-	-	-	-
**Distúrbios musculoesqueléticos e do tecido conjuntivo**														
Artralgia	27	0	-	-	-	-	-		10	0,0	3,15	1,44; 6,88	0,98	0,12
Dor em extremidade	186	0	-	-	-	-	-		46	6,5	1,76	1,26; 2,46	0,61	0,22
Dor nas costas	16	0	-	-	-	-	-		8	12,5	5,35	2,01; 14,26	1,25	0,25
Dor no pescoço	6	0	-	-	-	-	-		4	25	10,69	1,96; 58,40	-	-
Fraqueza muscular	28	8	25,0	3,75	1,65; 8,53	1,19	0,26		10	40,0	2,97	1,37; 6,44	0,94	0,08
Mialgia	290	57	5,3	2,23	1,73; 3,11	0,99	0,64		123	3,3	4,02	3,18; 5,09	1,40	1,14
**Distúrbios dos tecidos cutâneos e subcutâneos**														
Angioedema	81	0	-	-	-	-	-		20	0,3	1,76	1,06; 2,91	-	-
Prurido	596	112	6,3	2,22	1,80; 2,74	0,94	0,69		122	4,9	1,39	1,13; 1,70	0,37	0,13
Suor frio	5	3	0,0	14,06	2,35; 84,16	-	-		0	-	-	-	-	-
Urticária	240	0		-	-	-	-		55	9,1	1,60	1,18; 2,16	0,51	0,16
**Infecções e infestações**														
Faringite	6	0	-	-	-	-	-		4	0,0	10,69	1,96; 58,40	-	-
Pneumonia	13	0	-	-	-	-	-		5	100,0	3,34	1,09; 10,22	-	-
Rinite	156	35	0,0	2,73	1,87; 3,99	1,14	0,70		59	0,0	3,28	2,37; 4,54	1,21	0,85
**Distúrbios dos sistemas hematológico e linfático**														
Dor no linfonodo	6	0	-	-	-	-	-		5	0,0	26,74	3,12; 228,87	1,49	0,12
Linfadenopatia	73	0	-	-	-	-	-		41	4,9	6,90	4,34; 10,97	1,72	1,26
**Investigações**														
Pressão arterial diminuída	8	0	-	-	-	-	-		6	0,0	16,05	3,24; 79,53	1,51	0,27
**Distúrbios psiquiátricos**														
Ansiedade	6	4	25,0	18,75	3,43; 102,41	1,53	0,08		0	-	-	-	-	-
**Distúrbios cardíacos**														
Bloqueio atrioventricular de 1º grau	11	0	-	-	-	-	-		7	14,3	9,36	2,74; 32,00	1,44	0,32
Palpitações	10	3	0,0	4,02	1,04; 15,54	-	-		6	16,7	8,02	2,26; 28,44	1,32	0,13
**Distúrbios do sistema imunitário**														
Hipersensibilidade	216	36	5,6	1,88	1,31; 2,70	0,74	0,31		53	7,5	1,75	1,28; 2,39	0,61	0,24
**Distúrbios do ouvido e do labirinto**														
Vertigem	26	7	0,0	3,46	1,45; 8,23	1,08	0,10		8	25,0	2,38	1,03; 5,47	-	-

a Total de notificações na base de dados; ^b^Total de notificações por vacina (CoronaVac ou Pfizer); ^c^Percentual de eventos supostamente atribuíveis à vacinação ou imunização graves; ^d^Dado não aplicável para análise.

Para a vacina Sinovac, os termos hipersensibilidade e vertigem foram identificados por ambas as análises e estão descritos na bula. Para a vacina Pfizer, 3 termos foram identificados (bloqueio atrioventricular de primeiro grau, palpitações e hipersensibilidade), e apenas bloqueio atrioventricular não descrito em bula (11) (Tabela 3).

## Discussão

Este é o primeiro estudo nacional que apresenta resultados de Esavi monitorados pela farmacovigilância com a identificação de notificação desproporcional para vacinas de covid-19 aplicadas em crianças e adolescentes no Brasil. A maioria dos eventos detectados para as vacinas foram coincidentes em ambos os cálculos de desproporcionalidade e relacionados aos distúrbios respiratórios, torácicos e do mediastino. Entre os termos coincidentes, a maioria não estava descrita em bula e foi notificada como Esavi não grave e autolimitada. De forma geral, para vacina Sinovac os principais achados não descritos em bula foram: disartria, epistaxe, prurido ocular e fraqueza muscular. Em relação à vacina Pfizer, os eventos não descritos foram dor em linfonodo, epistaxe, bloqueio atrioventricular de 1º grau, hipersonia e dor torácica.

Este estudo apresenta uma limitação clássica de estudos caso-não-caso em bases de dados de vigilância passiva: a possibilidade de subnotificação de Esavi. Na farmacovigilância de rotina, a taxa de notificação representa, em média, de 6% a 10% dos casos reais de Esavi, sendo a subnotificação ainda maior para os casos não graves (17). Isso pode dificultar a mensuração da incidência real dos Esavi e limitar a sensibilidade da detecção de sinais por análise de desproporcionalidade (18). Este estudo também pode ter sofrido viés de notoriedade, que ocorre quando uma vacina recebe atenção da mídia sobre eventos adversos específicos, aumentando assim as notificações desses eventos (19). Além disso, a validade externa é limitada por fatores como diferenças nos calendários vacinais, práticas de notificação, cobertura vacinal e estrutura dos sistemas de vigilância em outros países. Tais limitações podem influenciar a representatividade dos eventos detectados, devendo os achados serem interpretados com cautela e complementados por outras fontes de evidência.

Atualmente, não há uma referência específica que determine quais limites devem ser adotados para definição de sinais de notificação desproporcional. Os limites comumente utilizados são estabelecidos buscando equilibrar duas questões: a possibilidade de gerar muitos “sinais falso-positivos” se o limite for muito baixo, ou de reduzir a identificação de “sinais potenciais” se o limite for muito alto (5,20).

De forma geral, a maioria dos sinais de notificação desproporcional identificados esteve relacionada a termos incluídos na classe de SOC “Distúrbios respiratórios, torácicos e do mediastino”, a saber: congestão nasal, dor orofaríngea, irritação na garganta, espirros, tosse e disfonia. Ao considerar esses potenciais eventos, é importante ressaltar que, em 2022, o maior número de doses administradas foi observado concomitantemente aos períodos de maior incidência de covid-19 nessa população. É provável que esses sinais de notificação desproporcional estejam relacionados aos casos de covid-19 e não propriamente às vacinas (21).

Os sinais de notificação desproporcional identificados para a vacina Sinovac estiveram distribuídos entre diferentes SOC e PT avaliados, a saber: calafrios, disartria, epistaxe, espirros, hiperemia ocular, prurido ocular, desconforto abdominal, fraqueza muscular, rinite e vertigem. Embora a epistaxe tenha sido identificada como sinal de notificação desproporcional para ambas as vacinas avaliadas, foram levantadas hipóteses relacionando-a a complicações da técnica de coleta com *swab* nasal para rRT-PCR de covid-19 (22). Ademais, vale destacar que a epistaxe apresenta maior incidência entre 7 e 13 anos de idade, e que sangramentos espontâneos, associados ao trauma digital, são as causas mais comuns de sangramento nasal na infância, geralmente de caráter benigno (23).

A disartria foi um termo identificado como sinal de notificação desproporcional e refere-se a um grupo de distúrbios resultantes de alterações no controle muscular dos mecanismos da fala, devido a danos no sistema nervoso central ou periférico. Há ampla variabilidade subjetiva na avaliação da disartria entre os profissionais de saúde (24). Atualmente, não há evidências que estabeleçam uma relação causal entre disartria e a vacina avaliada, contudo, sua detecção indica a necessidade de uma avaliação aprofundada dos casos para melhor compreensão do evento.

Ao observar os sinais de notificação desproporcional por tipo de vacina, é possível identificar que, para a vacina Pfizer, o termo “dor no peito”, apesar de não estar descrito especificamente na bula da vacina (11), pode ser sintoma de reações descritas como miocardite (25,26).

Outro termo identificado para a vacina Pfizer foi “bloqueio atrioventricular de primeiro grau”, detectado por ambas as medidas de desproporcionalidade e não descrito na bula do imunobiológico (11). Destaca-se, no entanto, que estudos populacionais de grande escala, realizados na Inglaterra e em Israel, constataram que a vacina da Pfizer não estava associada a maior risco de arritmias cardíacas ou bloqueios atrioventricular (26,27). Por outro lado, a infecção por SARS-CoV-2 foi associada a um aumento no risco de diversos eventos adversos cardíacos, como infarto do miocárdio, arritmias, miocardite e pericardite (26,27).

A identificação de dois sinais de notificação desproporcional com possível envolvimento cardíaco pode estar relacionado à maior sensibilidade dos profissionais de saúde em notificar tais eventos, diante da ampla divulgação, tanto científica quanto midiática, sobre o risco de miocardite e pericardite com vacinas de mRNA (25).

Entre os resultados, observaram-se termos cuja plausibilidade biológica com a vacina é limitada ou inexistente, o que reforça a necessidade de análise crítica quanto à qualidade das notificações. Parte desses achados poderia ser justificada pela ocorrência de erros de codificação, visto que o dicionário médico para atividades regulatórias (*medical dictionary for regulatory activities,* MedDRA) foi implementado no sistema de notificação de Esavi em 2021, e os treinamentos quanto à codificação pelo dicionário médico continuam em andamento.

As duas medidas de desproporcionalidade forneceram estimativas mais precisas, especialmente para relações com poucos casos relatados. Estudo anterior indica que, à medida que o número de notificações de um par vacina-Esavi aumenta, os métodos tendem a apresentar resultados semelhantes (28). Vale destacar que o método frequentista ROR gera mais sinais falso-positivos de notificação desproporcional, mas menos falso-negativos, enquanto os IC minimizam as relações falso-positivas (28). Alguns autores indicam que o ROR teria melhor desempenho do que outras técnicas no que se refere à detecção precoce de sinais (28,29).

No Brasil, a detecção de sinais de notificação desproporcional por métodos quantitativos ainda é uma ferramenta subutilizada e, atualmente, não está plenamente implementada na rotina do PNI. Assim, torna-se de suma importância a continuidade do monitoramento e da gestão de sinais de notificação desproporcional no âmbito do PNI, de forma a gerar evidências científicas sobre os eventos relatados de maneira sistematizada e auditável, o que contribuirá para uma vacinação segura e subsidiará discussões estratégicas na redução da hesitação vacinal.

Os resultados deste estudo refletem o perfil de notificações espontâneas de Esavi para as vacinas de covid-19 administradas em crianças e adolescentes no Brasil durante 2022. Foi possível identificar e descrever os sinais de notificações desproporcionais. Ressalta-se que análises quantitativas de dados de vigilância passiva, como as aqui apresentadas, têm como objetivo principal o levantamento de hipóteses pela comunidade científica, não sendo adequadas para inferência causal (5), o que indica a necessidade de estudos epidemiológicos adicionais para investigar cada sinal de notificação desproporcional apontado. Embora os resultados não permitam generalizações diretas, podem fornecer subsídios comparativos a outros para populações com características semelhantes em contextos de programas nacionais de imunizações que utilizam sistemas de farmacovigilância passiva. Por fim, há necessidade de aprimoramento no processo de codificação dos Esavi com o uso do dicionário médico para atividades regulatórias pelos profissionais notificadores no Brasil.

## Data Availability

Os dados agregados e anonimizados podem ser acessados em:https://doi.org/10.48331/SCIELODATA.5XCF1B.
